# The role of human microbiota in breast cancer pathogenesis and treatment

**DOI:** 10.1002/imt2.70142

**Published:** 2026-06-14

**Authors:** Jie Qiu, Xiaojia Wang, Man Li, Jing Guo, Shipeng Ning, Yi‐Zhou Gao, Xinyi Zhang, Xuli Meng, Yiding Chen, Yunxiang Zhou

**Affiliations:** ^1^ Department of Breast and Thyroid Surgery Shaoxing People's Hospital Shaoxing Zhejiang China; ^2^ School of Medicine Shaoxing University Shaoxing Zhejiang China; ^3^ Department of Breast Medical Oncology Zhejiang Cancer Hospital Hangzhou Zhejiang China; ^4^ Department of Breast Oncology The Second Hospital of Dalian Medical University Dalian Liaoning China; ^5^ Department of Microbiology and Immunology Stanford University School of Medicine Stanford California USA; ^6^ Department of Breast Surgery The Second Affiliated Hospital of Guangxi Medical University Nanning Guangxi China; ^7^ Department of Environmental Hygiene, School of Public Health Harbin Medical University Harbin Heilongjiang China; ^8^ Division of Solid Tumor Translational Oncology, German Cancer Consortium (DKTK), Partner Site Essen A Partnership Between German Cancer Research Center (DKFZ) and University Hospital Essen Essen Nordrhein‐Westfalen Germany; ^9^ Bridge Institute of Experimental Tumor Therapy, West German Cancer Center University Hospital Essen, University of Duisburg‐Essen Essen Nordrhein‐Westfalen Germany; ^10^ Cancer Center, Department of Breast Surgery Zhejiang Provincial People's Hospital Hangzhou Zhejiang China; ^11^ Department of Breast Surgery and Oncology, The Second Affiliated Hospital Zhejiang University School of Medicine Hangzhou Zhejiang China; ^12^ Cancer Institute (Key Laboratory of Cancer Prevention and Intervention, China National Ministry of Education), The Second Affiliated Hospital Zhejiang University School of Medicine Hangzhou Zhejiang China

## Abstract

The human microbiota contributes to breast cancer pathogenesis and treatment response through interactions involving the oral, gut, and breast microbial niches. (Left) Multi‐site dysbiosis is characterized by altered microbial composition and abundance across different anatomical sites. (Middle) Microbial components and metabolites influence tumor progression and the tumor microenvironment through mechanisms including immune, metabolic, and inflammatory regulation. (Right) The microbiota and their metabolites further affect therapeutic efficacy and toxicity in immunotherapy, chemotherapy, endocrine therapy, and human epidermal growth factor receptor 2 (HER2)‐targeted therapy, highlighting the translational potential of microbiota‐targeted strategies in breast cancer.

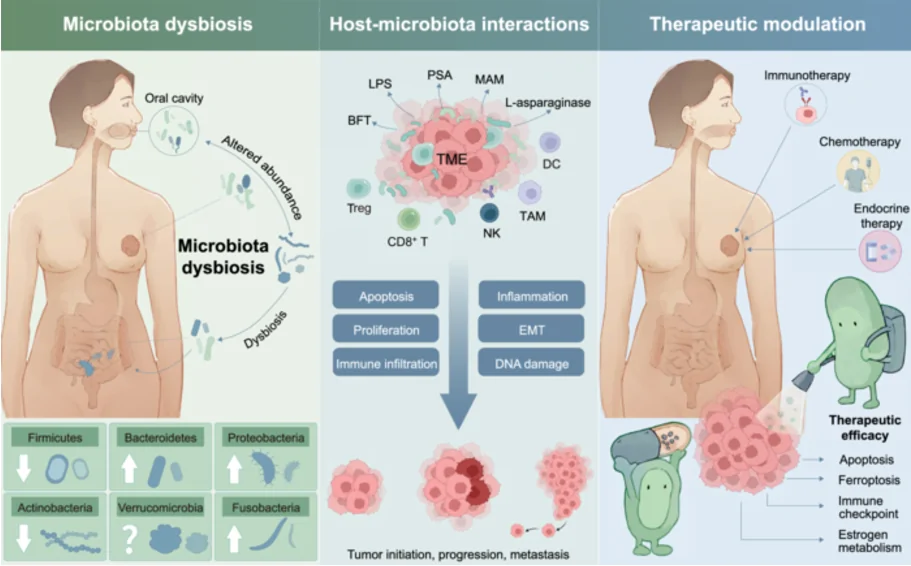

Breast cancer remains a leading cause of cancer‐related mortality in women worldwide [1]. Historically, the breast was considered a sterile organ. Recent studies have demonstrated that breast tissue harbors a low‐biomass microbiota with distinct compositional differences between healthy and malignant tissues [2]. These microorganisms may modulate the tumor microenvironment (TME) through interactions with epithelial, stromal, and immune cells. Oral and gut microbiota may further influence breast cancer biology through local and systemic crosstalk, thereby affecting disease initiation and therapeutic response (Figure 1) [2, 3]. Notably, tumor–microbiota interactions are relevant in breast cancer from a translational perspective, as both estrogen metabolism and systemic therapies (e.g., endocrine therapy and immunotherapy) may be modulated by the microbiota [2, 3]. Understanding these complex interactions offers opportunities to identify biomarkers, optimize current treatments, and develop microbiota‐targeted interventions, thereby potentially improving diagnosis, prognosis, and therapeutic precision in breast cancer management.

**Figure 1:**
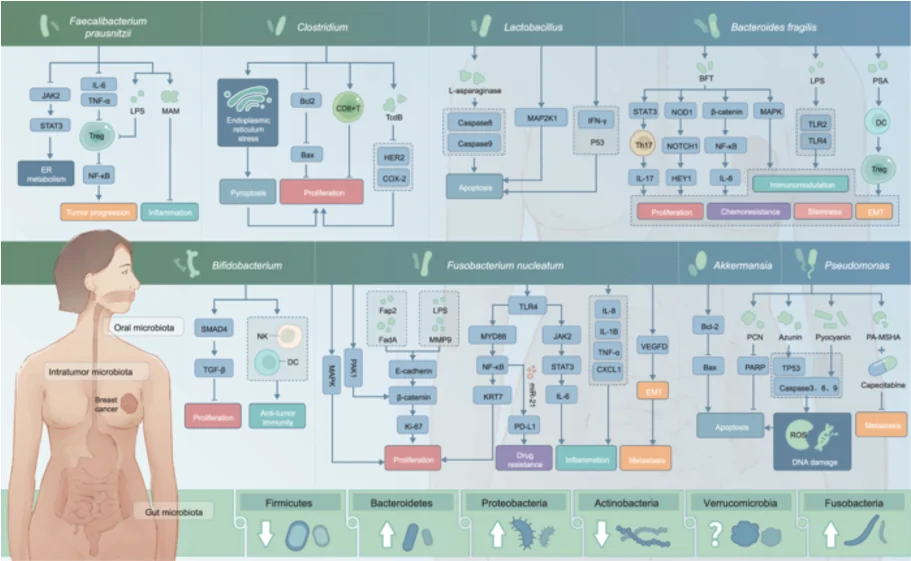
Crosstalk between the microbiota and the malignant progression of breast cancer. Microbiota from the gut, oral cavity, and intratumoral sites are implicated in breast cancer pathogenesis. Microbial phyla, such as Firmicutes (e.g., *Streptococcus*, *Staphylococcus*), Bacteroidetes (e.g., *Porphyromonas*), Proteobacteria (e.g., *Pseudomonas*, *Proteus*), Actinobacteria (e.g., *Propionibacterium*, *Corynebacterium*), Verrucomicrobia, and Fusobacteria (e.g., *Fusobacterium*), show altered relative abundance patterns between breast tumor and non‐tumor tissues (although inconsistencies remain across studies). These taxa influence tumor initiation, progression, and metastasis through distinct or overlapping molecular mechanisms, including regulation of apoptosis, proliferation, immune infiltration, inflammation, epithelial‐to‐mesenchymal transition (EMT), and DNA damage. BFT, *Bacteroides fragilis* toxin; DC, dendritic cell; ER, estrogen receptor; LPS, lipopolysaccharide; MAM, microbial anti‐inflammatory molecule; MMP9, matrix metalloproteinase‐9; NK, natural killer cell; NOD1, nucleotide‐binding oligomerization domain‐containing protein 1; PA‐MSHA, *Pseudomonas aeruginosa‐*mannose‐sensitive hemagglutinin; PARP, poly (ADP‐ribose) polymerase; PCN, phenazine‐1‐carboxamide; PSA, polysaccharide A; ROS, reactive oxygen species; TcdB, *Clostridioides difficile* toxin B; Treg, regulatory T cell; TLR, Toll‐like receptor; VEGFD, vascular endothelial growth factor‐D.

## BREAST MICROBIOTA DYSBIOSIS IN BREAST CANCER

The human breast microbiota is distinct from that of other organs, although a fully defined core profile has not yet been established. Proteobacteria is the dominant phylum in breast tissue, with additional contributions from Firmicutes, Actinobacteria, and Bacteroidetes [2]. In breast cancer, the microbial composition of tumor tissue differs significantly from that of normal tissue (Figure 1) [4]. Subtype‐specific microbial patterns have also been reported, including lower abundance of genera within the class Alphaproteobacteria in hormone receptor‐positive tissues, although such patterns are largely inconsistent across studies [5]. Beyond compositional alterations, emerging evidence suggests that breast microbiota dysbiosis is closely linked to the local immune microenvironment. Notably, *Propionibacterium* and *Staphylococcus*, both depleted in tumor tissue, show inverse associations with oncogenic immune features. Moreover, *Propionibacterium* and *Streptococcus* correlate positively with T‐cell activation‐related genes [4]. These findings support a potential role for the breast microbiota in shaping tumor–immune interactions and influencing breast cancer progression.

## EXTRAMAMMARY MICROBIOTA AND BREAST CANCER PATHOGENESIS

Beyond the local breast microbiota, accumulating evidence suggests that extramammary microbial niches contribute to breast cancer development and progression, with the gut microbiota being the most extensively investigated. In the healthy intestine, Firmicutes and Bacteroidetes are the dominant phyla and contribute to intestinal homeostasis. Gut microbial dysbiosis, often characterized by altered Firmicutes/Bacteroidetes balance, decreased diversity, and expansion of potentially pathogenic taxa, may promote tumorigenesis through enterotoxin production and chronic inflammation [6, 7]. For instance, enterotoxigenic *Bacteroides fragilis* secretes *Bacteroides fragilis* toxin‐1 (BFT‐1), which binds to nucleotide‐binding oligomerization domain‐containing protein 1 (NOD1) in breast cancer cells, stabilizes NOD1, and activates Notch homolog 1 (NOTCH1) signaling pathway, thereby promoting breast cancer cell stemness and chemoresistance [7]. Notably, not all gut microbiota alterations observed in breast cancer are pro‐tumorigenic. *Faecalibacterium prausnitzii*, a Firmicutes member depleted in breast cancer patients, has been associated with anti‐tumor effects through inhibition of IL‐6 secretion and JAK2/STAT3 signaling [6]. In addition, gut microbiota may influence breast cancer pathogenesis by modulating estrogen deconjugation and enterohepatic recycling [8].

Compared with the gut microbiota, evidence linking oral microbiota to breast cancer remains limited. *Fusobacterium nucleatum*, a common oral microorganism, has been detected in breast tumors and is associated with tumor growth and metastatic progression. Mechanistically, *Fusobacterium nucleatum* binds tumor‐displayed Gal‐GalNAc via its Fap2 lectin, promoting breast tumor colonization. It subsequently suppresses the accumulation of tumor‐infiltrating T cells and drives tumor growth and metastasis, and these effects can be counteracted by antibiotic treatment [3]. These findings suggest that oral microbiota may also contribute to breast cancer pathogenesis, although further mechanistic and clinical studies are needed.

## MICROBIOTA‐DERIVED METABOLITES AND BREAST CANCER PATHOGENESIS

Beyond direct host–microbiota interactions, accumulating evidence suggests that microbiota‐derived metabolites play multifaceted roles in breast cancer progression (Table S1). For instance, secondary bile acids, produced by the gut microbiota, including *Clostridium* species, exert divergent effects in breast cancer: lithocholic acid suppresses tumor cell proliferation, inhibits the production of vascular endothelial growth factor, and promotes antitumor immunity, whereas deoxycholic acid has been implicated as a procarcinogenic agent [9]. Although some studies have implicated the gut microbial metabolite trimethylamine N‐oxide in promoting tumor progression, Wang et al. found that it can suppress triple‐negative breast cancer by inducing endoplasmic reticulum stress‐mediated pyroptosis, which subsequently activates CD8^+^ T cell antitumor immunity [10]. Enterolactone is a gut microbiota‐derived metabolite of dietary flaxseed lignans. In breast cancer, enterolactone suppresses tumor progression by downregulating CD38, a key immunosuppressive molecule, thereby partially reversing the immunosuppressive TME [11]. Altered short‐chain fatty acid (SCFA) profiles have been observed in breast cancer, reflecting both microbiota dysbiosis and host metabolic reprogramming, with individual metabolites exerting context‐dependent effects on tumor biology [9, 12]. Notably, butyrate, among these SCFAs, can enhance cytotoxic CD8^+^ T‐cell responses via an epigenetic mechanism that promotes IL‐12 signaling, thereby strengthening antitumor immunity [12]. Other metabolites, including urolithin A, have also been implicated in modulating breast cancer progression [13]. Collectively, microbiota‐derived metabolites represent an important mechanistic link between microbial dysbiosis and breast cancer progression, and may serve as potential predictive and prognostic biomarkers.

## MICROBIOTA AND IMMUNOTHERAPY RESPONSE IN BREAST CANCER

**Figure 2:**
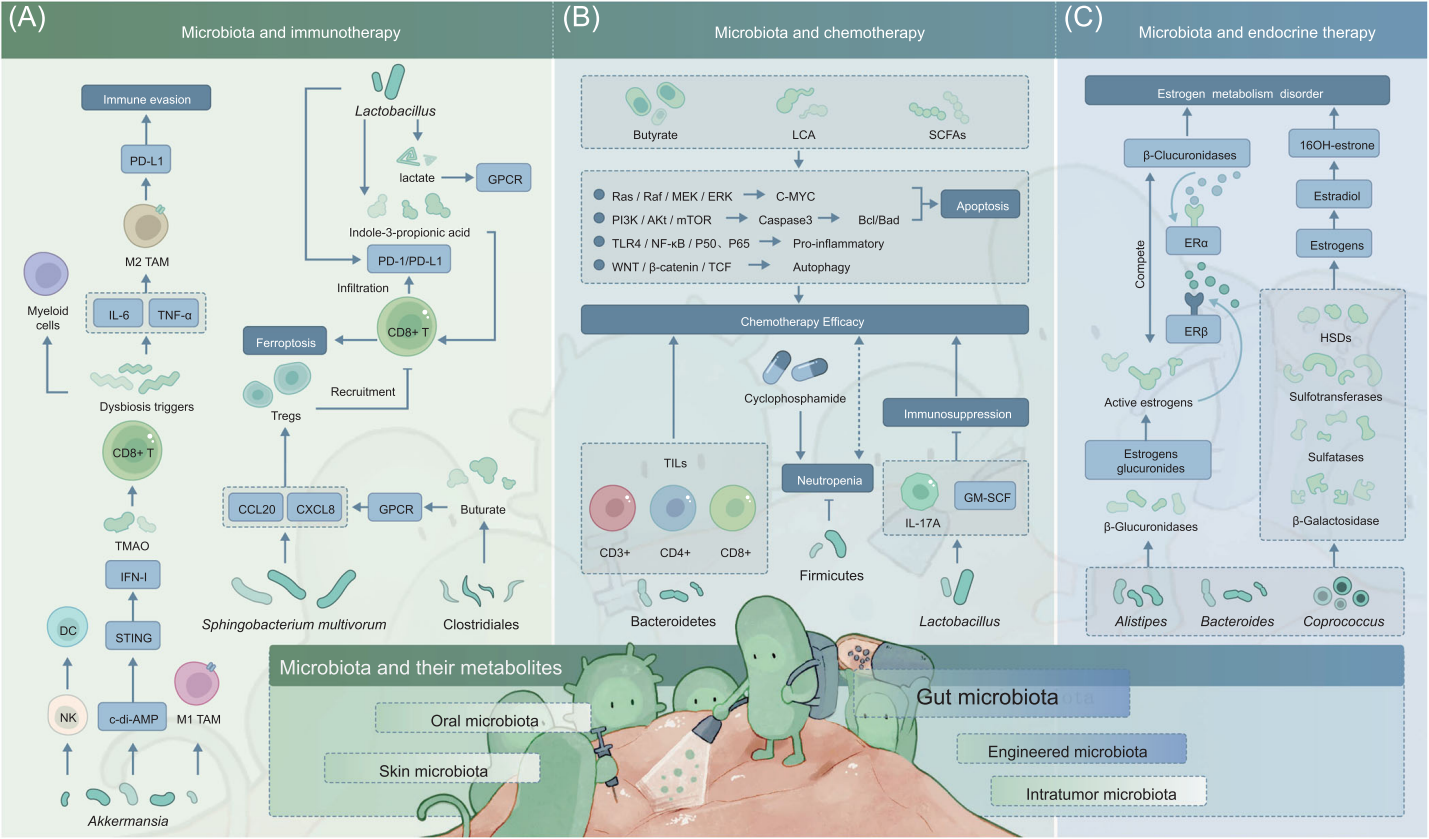
Microbiota and their metabolites in breast cancer therapy. (A) Microbiota‐derived metabolites and bacterial taxa modulate immune responses and influence the efficacy of immune checkpoint blockade in immunotherapy. (B) Microbiota composition and metabolites affect chemotherapy efficacy and toxicity through regulation of apoptosis, autophagy, immune cell infiltration, and inflammatory responses. (C) Microbial enzymes, particularly β‐glucuronidase, regulate estrogen deconjugation and enterohepatic circulation, thereby modulating responsiveness to endocrine therapy. Representative microbial taxa and metabolites are mapped across the three therapeutic contexts, highlighting their potential roles in modulating treatment response. c‐di‐AMP, cyclic di‐adenosine monophosphate; DC, dendritic cell; ER, estrogen receptor; GM‐CSF, granulocyte macrophage colony‐stimulating factor; GPCR, G‐protein coupled receptor; HSDs, hydroxysteroid dehydrogenases; LCA, lithocholic acid; NK, natural killer cell; SCFAs, short‐chain fatty acids; STING, stimulator of interferon genes; TAM, tumor‐associated macrophage; TILs, tumor‐infiltrating lymphocytes; TMAO, trimethylamine N‐oxide; Treg, regulatory T cell.

Accumulating evidence supports a role for microbiota in modulating therapeutic responses in breast cancer (Figure 2). Among these, immunotherapy has attracted considerable attention (Figure 2A). In particular, both microbiota‐derived metabolites (e.g., enterolactone) and compositional shifts (e.g., *Akkermansia* enrichment) have been associated with enhanced responsiveness to PD‐1/PD‐L1 blockade in breast cancer [11]. In contrast, intratumoral *Sphingobacterium multivorum* reduces the efficacy of anti‐PD‐1 therapy by promoting the recruitment of regulatory T cells via chemokines (e.g., CCL20) and decreasing CD8^+^ T‐cell infiltration [14]. Collectively, these findings highlight the therapeutic potential of microbiota‐directed strategies for enhancing immunotherapy efficacy.

## MICROBIOTA AND TARGETED THERAPY RESPONSE IN BREAST CANCER

Gut microbiota may also influence the efficacy of human epidermal growth factor receptor 2 (HER2)‐targeted therapy in breast cancer. In HER2‐positive breast cancer, disruption of gut microbiota by antibiotic treatment impairs trastuzumab antitumor activity and is associated with reduced dendritic‐cell activation, decreased IL‐12p70 production, and diminished recruitment of activated CD4^+^ T cells within the TME [15]. Consistently, trastuzumab‐nonresponsive patients exhibit lower gut microbial diversity and reduced abundance of several commensal bacterial families compared with responders [15]. These findings suggest that gut microbiota may modulate HER2‐targeted therapy responsiveness, warranting further investigation to elucidate underlying mechanisms and clinical implications.

## MICROBIOTA AND CHEMOTHERAPY RESPONSE AND TOXICITY IN BREAST CANCER

Microbiota may play a role in modulating chemotherapy outcomes (Figure 2B). The gut microbiota contributes to chemotherapy efficacy and treatment tolerance through regulation of drug metabolism and host immune responses. Ma et al. observed that enterotoxigenic *Bacteroides fragilis* was enriched in breast tumors of patients resistant to taxane‐based neoadjuvant chemotherapy, and further demonstrated in mouse models and cell lines that enterotoxigenic *Bacteroides fragilis* and its secreted toxin BFT‐1 contribute to resistance to chemotherapy [7]. In terms of toxicity, a prospective cohort of 301 breast cancer patients demonstrated that lower baseline gut microbial α‐diversity and enrichment of specific Firmicutes‐related taxa, including class Bacilli A and order Paenibacillales, are associated with a higher risk of severe hematological toxicity, whereas higher abundance of phylum Synergistota is associated with a reduced risk of severe neutropenia [16]. Collectively, these findings suggest that the gut microbiota has potential as a biomarker and therapeutic target in breast cancer chemotherapy management.

## MICROBIOTA AND ENDOCRINE THERAPY RESPONSE IN BREAST CANCER

For patients with estrogen receptor‐positive breast cancer, microbiota‐mediated estrogen metabolism may play an important role in endocrine therapy response (Figure 2C). Bacterial β‐glucuronidase enzymes catalyze the deconjugation of estrogens in the intestine, facilitating their reabsorption via enterohepatic circulation and thereby influencing systemic estrogen exposure and endocrine responsiveness [8]. Beyond the gut axis, evidence also suggests potential local estrogen reactivation within the breast microenvironment. Genes encoding β‐glucuronidase enzymes are enriched in nipple aspirate fluid from breast cancer patients, suggesting an increased capacity for conjugated estrogen reactivation [9]. Functionally, enhanced estrogen reactivation may promote tumor proliferation, metabolic reprogramming, epithelial–mesenchymal transition, and therapy resistance, including tamoxifen resistance [9]. Collectively, these findings suggest that microbiota‐mediated estrogen metabolism may contribute to endocrine therapy response heterogeneity in breast cancer, highlighting its potential for treatment stratification.

## MICROBIOTA‐TARGETED THERAPEUTIC STRATEGIES IN BREAST CANCER

Microbiota‐targeted interventions in breast cancer can be broadly categorized into microbiota modulation, microbiota depletion or reshaping, and engineered microbiota‐based platforms [17, 18]. Among these, microbiota modulation (e.g., probiotics and dietary interventions) represents the most accessible and safest strategy, but with relatively limited clinical efficacy evidence [17, 18, 19, 20]. Microbiota reshaping approaches, particularly fecal microbiota transplantation (FMT), have shown promising therapeutic potential in preclinical models, especially in combination with immune checkpoint blockade and HER2‐targeted therapy [17]. In contrast, antibiotic‐induced microbiota depletion remains controversial because of its potential to impair antitumor immunity and disrupt microbial homeostasis [17]. To overcome the limitations of conventional depletion, nanotechnology‐based platforms offer precision approaches [17]. Engineered nanovehicles can selectively target pro‐tumorigenic bacteria while preserving commensal microbiota. Furthermore, bacterial–nanomaterial hybrids and oncolytic viruses (e.g., T‐VEC or MV‐s‐NAP) enable delivery of therapeutic cargo into hypoxic tumor regions, potentially overcoming microenvironmental barriers and broadening the therapeutic scope of microbiota‐based strategies [17, 18].

Despite this substantial therapeutic potential, microbiota‐based interventions in breast cancer remain largely confined to preclinical and early exploratory clinical stages [17, 19]. Ongoing trials are evaluating probiotics for immune modulation (NCT03358511) and engineered viral or microbial platforms for safety and feasibility (NCT04521764), yet clinical translation still lags behind other malignancies, such as melanoma, where FMT combined with anti‐PD‐1 therapy has achieved objective response rates of up to 65% in a multicenter phase I trial [19]. Key barriers include safety concerns (e.g., uncontrolled microbial expansion, mutation, and horizontal gene transfer), as well as substantial interindividual variability in baseline microbiota composition and dietary background [17, 18, 19]. Future development will likely depend on personalized microbiota‐based stratification and integrative “One Health” frameworks, together with precision‐engineered microbial or nanotechnology‐based systems enabling biomarker‐guided therapeutic delivery [17, 18].

## SHORT SUMMARY

Increasing evidence supports an association between the human microbiota and breast cancer biology across breast, gut, and oral microbial niches. These microbial communities and their metabolites may influence tumor progression and the tumor microenvironment through immune‐, metabolic‐, and inflammation‐related pathways. Emerging evidence suggests potential links between microbiota composition and responses to systemic therapies, including immunotherapy, HER2‐targeted therapy, chemotherapy, and endocrine therapy, although current data remain largely observational and heterogeneous. To date, no microbiota‐based biomarker or intervention has been validated for routine clinical use in breast cancer. Future studies should integrate mechanistic investigations with translational research to clarify causal relationships and clinical relevance.

## AUTHOR CONTRIBUTIONS

**Jie Qiu**: Conceptualization; writing—original draft. **Xiaojia Wang**: Supervision; writing—review and editing. **Man Li**: Writing—review and editing; methodology. **Jing Guo**: Writing—original draft; data curation. **Shipeng Ning**: Writing—review and editing. **Yi‐Zhou Gao**: Writing—review and editing; data curation. **Xinyi Zhang**: Writing—original draft; visualization. **Xuli Meng**: Writing—review and editing. **Yiding Chen**: Funding acquisition; supervision; writing—review and editing. **Yunxiang Zhou**: Conceptualization; data curation; supervision; project administration; writing—review and editing; funding acquisition.

## CONFLICT OF INTEREST STATEMENT

The authors declare no conflicts of interest.

## ETHICS STATEMENT

No animals or humans were involved in this study.

## Supporting information


**Table S1:** Representative microbial taxa and microbiota‐derived metabolites implicated in breast cancer progression and therapeutic response.

## Data Availability

Data sharing is not applicable to this article, as no datasets were generated or analyzed during the current study. No new data and scripts were generated in this Commentary. Supplementary information (tables, graphical abstract, slides, videos, Chinese translated version, and updated materials) may be found in the online DOI or iMeta Science https://www.imeta.science/.
